# A New Method to Characterize Conformation-Specific Antibody by a Combination of Agarose Native Gel Electrophoresis and Contact Blotting

**DOI:** 10.3390/antib11020036

**Published:** 2022-05-12

**Authors:** Teruo Akuta, Toshiaki Maruyama, Chiaki Sakuma, Masataka Nakagawa, Yui Tomioka, Kevin Entzminger, Jonathan K. Fleming, Ryo Sato, Takashi Shibata, Yasunori Kurosawa, C. J. Okumura, Tsutomu Arakawa

**Affiliations:** 1 Research and Development Division, Kyokuto Pharmaceutical Industrial Co., Ltd., 3333-26, Aza-Asayama, Kamitezuna, Takahagi-shi 318-0004, Ibaraki, Japan; c.sakuma@kyokutoseiyaku.co.jp (C.S.); m.nakagawa@kyokutoseiyaku.co.jp (M.N.); y.tomioka@kyokutoseiyaku.co.jp (Y.T.); r.satou2@kyokutoseiyaku.co.jp (R.S.); t.shibata@kyokutoseiyaku.co.jp (T.S.); y.kurosawa@kyokutoseiyaku.co.jp (Y.K.); 2 Abwiz Bio Inc., 9823 Pacific Heights Blvd., Suite J, San Diego, CA 92121, USA; toshi@abwizbio.com (T.M.); kevinem@abwizbio.com (K.E.); jfleming@abwizbio.com (J.K.F.); cj@abwizbio.com (C.J.O.); 3 Alliance Protein Laboratories, 13380 Pantera Rd, San Diego, CA 92130, USA

**Keywords:** native gel, blotting, conformation-specific, antibody, SARS-CoV-2, conformation-indifferential

## Abstract

In this study, we review the agarose native gel electrophoresis that separates proteins and macromolecular complexes in their native state and transfer of the separated proteins from the agarose gel to membranes by contact blotting which retains the native state of these structures. Green fluorescent protein showed functional state both on agarose gel and blotted membrane. Based on the combined procedures, we discovered conformation-specific monoclonal antibodies against PLXDC2 and SARS-CoV-2 spike protein.

## 1. Introduction

Antibodies are important tools in biological studies and are also effective therapeutic drugs [1,2,3]. They are used to detect antigens in various extracts and intact cells in biological studies and, in the therapeutic field, to inhibit or mediate protein-protein interactions associated with human diseases. Antibodies may be formally classified into three groups. The first group binds only to the denatured proteins that may occur upon western blotting from sodium dodecylsulfate-polyacrylamide gel electrophoresis (SDS-PAGE). The second group binds only to the native or folded structures, called conformation-specific antibodies. Lastly, the third group binds to both structures and does not distinguish the structural differences, thus termed “conformation-indifferential”. Those antibodies that bind to the folded structure of antigen proteins (i.e., group two and three) are screened against structured proteins or macromolecular complexes, either native proteins or certain unique altered structures, e.g., amyloid structures [2,4,5,6,7,8]. Technology used for such a screening includes, but is not limited to, enzyme-linked immuno assay [9], dot blot [10], surface plasmon resonance [11], isothermal titration calorimetry [12], lateral flow immunoassay [13], and latex agglutination assay [14]. In in vitro or in vivo bioassays using various cells and tissues, antibodies are used as inhibitors for protein-protein interactions or as agonistic ligands (as the ‘gold standard’). Among these antibodies that recognize the native protein, antibodies belonging in group two are conformation-specific and hence do not bind to the denatured structures. Such conformation-specific antibodies are useful in distinguishing native functional antigens from the denatured antigens. Here, we have developed a new method to screen conformation-specific antibodies based on our novel agarose native gel electrophoresis and contact blotting, which facilitates retention of native structure on the blotted membranes. A similar blotting technique, called “diffusion blotting”, has been described for protein transfer from SDS-PAGE [15]. Similarly, a native gel electrophoresis system is commercially available using polyacrylamide gels. This vertical gel works well for acidic proteins, with electrical polarity set identical to sodium dodecyl sulfate-polyacrylamide gel electrophoresis. In theory, the same system should work for basic proteins by reversing the electrical polarity, but were found to be problematic due to band smearing [16,17]. Using agarose gels was found to reduce smears [17], but still not to be optimal, as seen by round band shape, although it generally works well for acidic proteins and their complexes [18,19,20,21,22,23,24,25,26,27,28,29]. Thus, we have undertaken to develop agarose native gel electrophoresis that works for any proteins and their complexes. Native electrophoresis using agarose gels has been extensively used for the analysis of nucleic acids and proteins in submarine mode of horizontal gels. It was suggested that placing the sample wells at the center of agarose gel should make simultaneous analysis of acidic and basic proteins or their complexes possible [30]. We review our recent development of technologies to identify conformation-specific and conformation-indifferential antibodies based on the agarose native gel electrophoresis developed as above and blotting of native proteins from agarose gels to membrane under the native conditions.

### 1.1. Antibody Production

**Figure 1:**
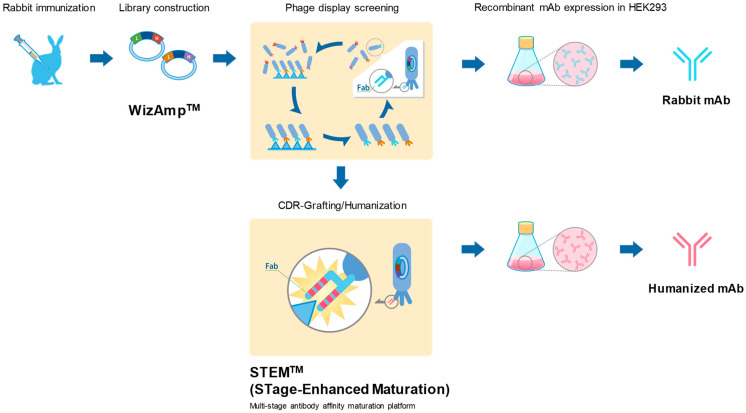
Overview of antibody production procedure.

We have used a general scheme depicted in Figure 1 to generate rabbit-derived monoclonal antibodies. In general, New Zealand White rabbits were immunized with an antigen, which can be peptides that are chemically synthesized, recombinant proteins, or whole cells. The antibody libraries were constructed from the B cells of the immunized rabbits and used to screen and isolate specific clones using phage display method as previously described [16]. Antibody libraries were constructed using the antibody gene cloning method, “WizAmp^TM^”, as described in a published patent (US 9,890,414). Antibodies were produced with HEK293 transient expression system using pTT5 vectors harboring cloned L and H antibody chains (National Research Council of Canada, Ottawa, ON, Canada). If required, the rabbit antibodies discovered were humanized by CDR-grafting and further engineered to obtain broader specificities with Abwiz Bio’s multi-stage antibody affinity maturation platform^TM^ (STage-Enhanced Maturation, Abwiz Bio Inc., San Diego, CA, USA).

Homogenized spleen and bone marrow were isolated from the immunized rabbits to construct the antibody library. Next, the antibody library was selected on antigen using phage display technology, which generated high affinity rabbit antibodies. Rabbit antibodies possess unique characteristics that confer high affinity, typically with dissociation constants in the range of the pico molar. Antibodies generated by the rabbit immune system undergo extensive hypermutation and even somatic gene conversion, a mechanism lacking in human and mouse immune systems [31]. In addition, rabbit antibodies have a longer light and heavy chain CDR3 (complement determining region) lengths and greater sequence diversity, which allows for sensitive and accurate detection of post-translational modifications that have been difficult to detect by antibodies from sources other than rabbits. Finally, the mature antibody was recombinantly produced with HEK293 transient expression system using pTT5 vectors harboring cloned L and H antibody chains.

#### 1.1.1. Anti-PLXDC2 Rabbit Monoclonal Antibody

For rabbit immunization, a whole cell antigen was used. The human PLXDC2 expressing cells were prepared by the transfection of HEK293 cells with pTT5 vector containing the entire PLXDC2 gene. Specific antibodies were screened and selected by flow cytometry using HEK293 cells expressing human PLXDC2.

#### 1.1.2. Anti-SARS-CoV-2 Spike RBD Humanized Monoclonal Antibody

For rabbit immunization, a recombinant protein was used. In brief, recombinant SARS-CoV-2 Spike RBD (receptor binding domain) protein was prepared by HEK293 cells. The anti-SARS-CoV-2 neutralizing antibodies against Alpha, Beta, Gamma, Kappa, Lambda, Mu, Delta, and Delta plus (and Omicron) variants were selected by using the recombinant purified antigen. Monoclonal antibodies discovered were then humanized by CDR-grafting and further engineered to obtain broader specificities.

### 1.2. Agarose Native Gel Electrophoresis

**Figure 2:**
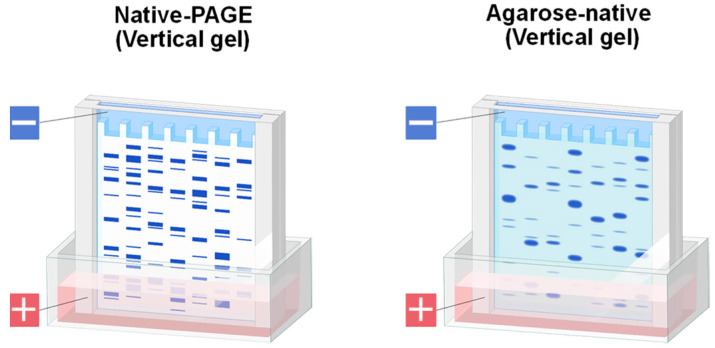
Schematic illustration of vertical gel. Left panel, commercially available native-PAGE. Right panel, agarose gels in vertical mode were prepared using commercially available empty cassette (product details, see [17]).

There are several native gel electrophoresis systems. One of the typical systems is a commercially available native polyacrylamide gel electrophoresis (N-PAGE) based on polyacrylamide gels and Tris-Glycine buffer system at pH of 8.1–8.3. As shown in Figure 2, it is cast into a vertical cassette and designed to perform well for acidic proteins (e.g., proteins with the isoelectric point, pI, below 8.1–8.3). Namely, they are negatively charged at those pH and migrate toward the anode placed at the bottom side of the gel. Other systems with agarose gels or polyacrylamide gels at different pHs have also been used.

**Figure 3:**
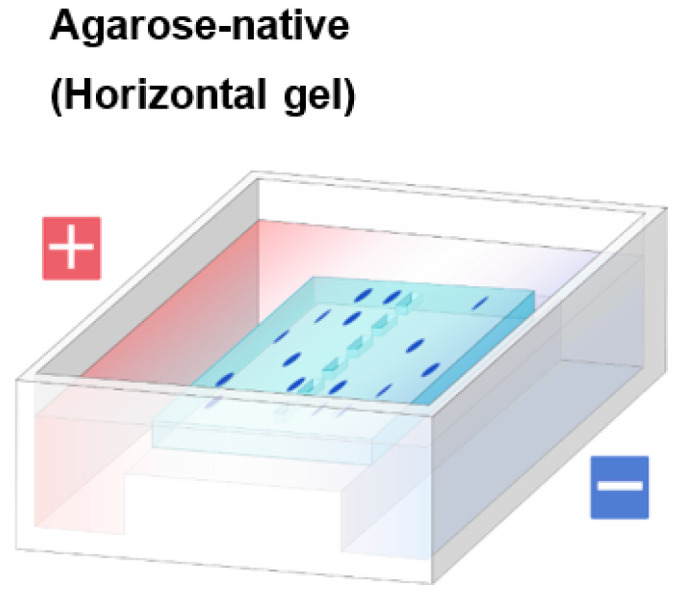
Schematic illustration of horizontal gel. Agarose gels in horizontal mode were prepared on Mupid flat tray.

**Figure 4:**
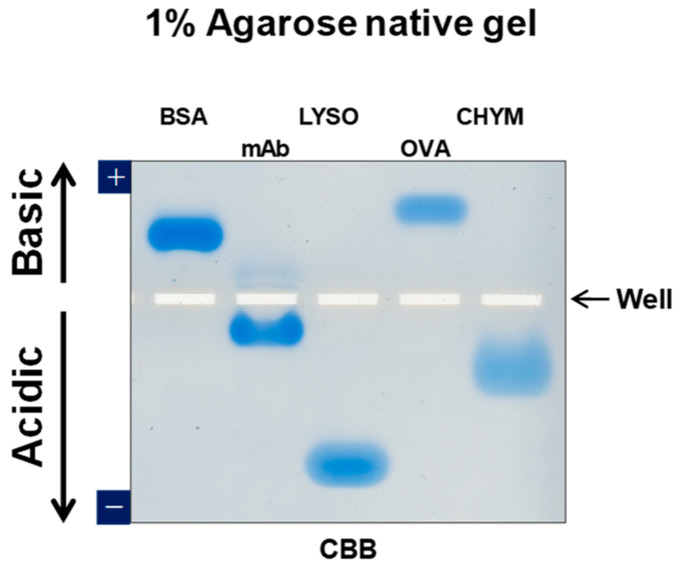
Typical data of agarose native gel electrophoresis. Five proteins were analyzed by agarose native gel electrophoresis. 1% UltraPure agarose was used and stained by CBB. Arrows show direction of acidic or basic proteins to migrate. Samples were loaded in the center position (marked Well).

We have developed an agarose-based system in both vertical mode as in Figure 2 and horizontal mode shown in Figure 3. Detail procedure of agarose native gel electrophoresis in vertical mode has been described [32], In brief, we mainly used empty cassette from Thermo Fisher Scientific or ATTO, which has been described in detail [32]. In this review, we will mainly focus on the use of horizontal (flat) gels, based on Mupid electrophoresis apparatus as shown in Figure 3. The running buffer we adopted for agarose gels was developed by McLellan for polyacrylamide gels [32,33] and is composed of 0.1 M His/0.1 M MES, pH 6.1 [34,35,36,37,38,39]. Lower concentration of equimolar His and MES at pH 6.1 may work, but often give inconsistent results [34]. Higher concentrations occasionally work better, albeit with limited improvement. Agarose commonly used was UltraPure agarose from Thermo Fisher Scientific. When necessary, we also used high resolution MetaPhor agarose from Lonza to achieve better size separation. Agarose was dissolved with hot 0.1 M His/0.1 M MES buffer at pH 6.1. Samples were mixed with the sample buffer made of the above buffer. Figure 4 shows the Coomassie Brilliant Blue (CBB) staining of model proteins run on 1% UltraPure agarose gel. The samples were loaded at the center well position. After electrophoresis, proteins were stained by Quick CBB PLUS (FUJI-FILM Wako Pure Chemical).These five proteins migrated according to their isoelectric points and hence charged states. Negatively charged (i.e., acidic) bovine serum albumin (BSA) and ovalbumin (OVA) migrated toward the anode, while the positively charged (basic) monoclonal antibody (mAB), lysozyme (LYSO) and chymotrypsin (CHYM) migrated toward the cathode at the electrophoresis running pH of 6.1. To probe their structure states, zymography may be used as a functional assay for lysozyme and chymotrypsin. Although not an absolute proof for function, we have developed a staining method for the unfolded structure, using SYPRO Orange fluorescence dye (Thermo Fisher Scientific, Waltham, MA, USA) [39]. We have observed that this dye binds only to the unfolded structure. Although not shown here, these five proteins could not be stained by this dye diluted 5000-fold with 50 mM Tris-HCl, pH 7.4 (neutral SYPRO Orange staining solution), which stained the unfolded structures on agarose gels, suggesting their folded structures.

### 1.3. Potential Applications of Native Proteins on Agarose Gel

**Figure 5:**
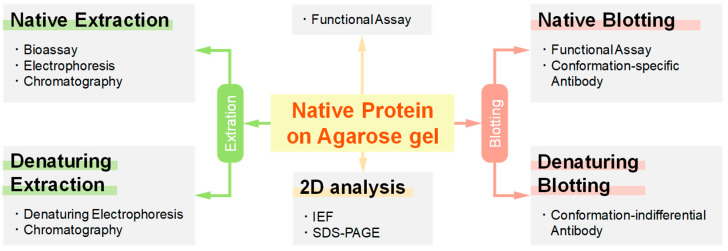
Schematic illustration of several application of agarose native gel electrophoresis. IEF, isoelectric focusing.

UltraPure agarose gels are highly porous as described above and thereby have potential application due to its porous nature. Such application is summarized in Figure 5. One application is based on ease of extraction from the porous agarose gel [32,35]. Native extraction can be used to analyze the activity of the proteins (e.g., binding properties of the extracted antibodies or antigens). Denaturing extraction as well as native extraction can be followed by another electrophoresis or chromatographic analysis. Native proteins on agarose gels can be directly assayed by diffusing a substrate through the porous agarose gel (zymography). By placing a strip of agarose gel on top of the 2nd dimension gel, 2D gel electrophoresis can be readily carried out [32]. Finally, more importantly such porous agarose gel may make blotting more efficient.

**Figure 6:**
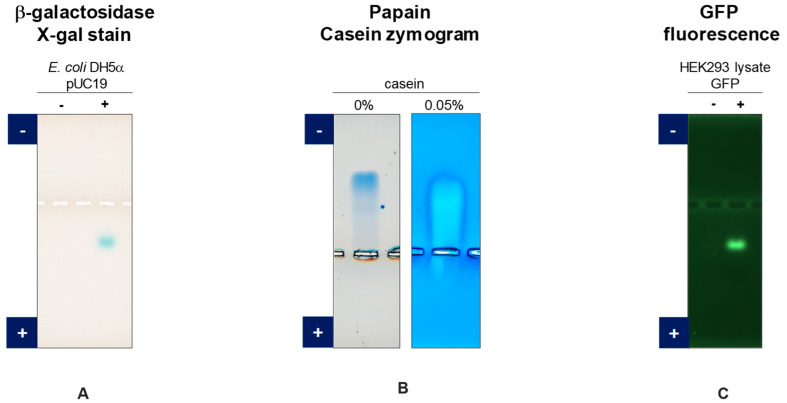
Zymography of enzymes and GFP fluorescence separated by agarose native gel electrophoresis. (**A**) Expression of β-galactosidase in *E. coli* DH5α without (−) and with (+) pUC19 expression. (**B**) Papain zymography. Left, CBB staining. Right, zymogram with casein as a substrate. (**C**) GFP fluorescence in HEK293 cell lysates without expression (−) and with expression (+).

Agarose native gel electrophoresis suites perfectly for zymography experiments of enzymes [40]. Native structure of enzymes separated as above should be retained and the His/MES buffer system is unlikely to interfere with the functional activity of most enzymes. Zymography can be performed by soaking the gel with the colorimetric substrates as exemplified below with a couple of enzymes. Figure 6 shows the activity of the proteins after gel electrophoresis: note that these data were assembled from the original data to simplify the point. Original figures are found in each reference publication [32,36].

One of the examples is an enzyme, β-galactosidase. An *E. coli* DH5α strain (LacZΔ M15) expresses inactive form of β-galactosidase due to the absence of α-peptide described below [41]. An *E. coli* harboring a plasmid pUC19, which encodes LacZα-peptide (110 amino acids), expresses both α-peptide and inactive β-galactosidase to generate a functional complex (α-complementation). As shown in Figure 6A, the expressed protein was analyzed by agarose native gel electrophoresis with the gel soaked with the substrate, X-gal. This substrate is converted to the greenish product by the active complex of α-peptide and inactive β-galactosidase (lane+). In the absence of pUC19 plasmid (lane−), there is no active β-galactosidase.

In another example, zymography with papain (pI = 8.75, 23 kDa), a cysteine protease, is attempted [42]. The papain is subjected to agarose native gel electrophoresis with 1% agarose containing 0.05% casein as a substrate at 1 µg papain/lane and stained with CBB, showing a band migrating toward the cathode (Figure 6B). During the electrophoresis, the active papain digests the surrounding substrate (i.e., 0.05% casein) while migrating through the gel, generating a white faded band. These results demonstrate that functional enzymes are retained on the agarose gels.

Green fluorescent protein (GFP) exhibits fluorescence only when it is native and folded. It was expressed in HEK293 cells as a soluble form and separated by agarose native gel electrophoresis. As shown in Figure 6C, a green fluorescent band can be clearly seen, indicating that it has a folded structure.

## 2. Blotting Methods

**Figure 7:**
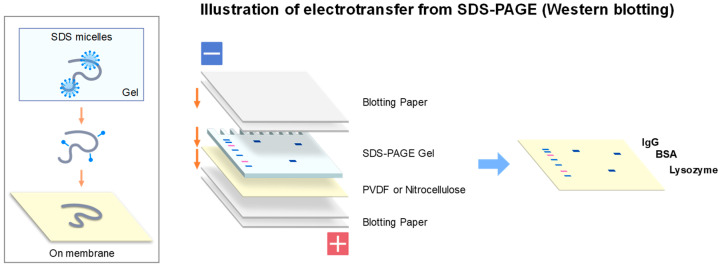
Schematic illustration of electroblotting from SDS-PAGE (original western blotting). SDS micelles bind to a polypeptide chain (inset, top) and may at least partially dissociate during transfer process (middle) and fully dissociate upon transfer to the membrane (bottom). Normal setup of electroblotting of SDS-PAGE gel is depicted in right side. Typical condition of blotting (semi-dry) was at a constant current of 1.5 mA/cm^2^ for 30 min.

**Figure 8:**
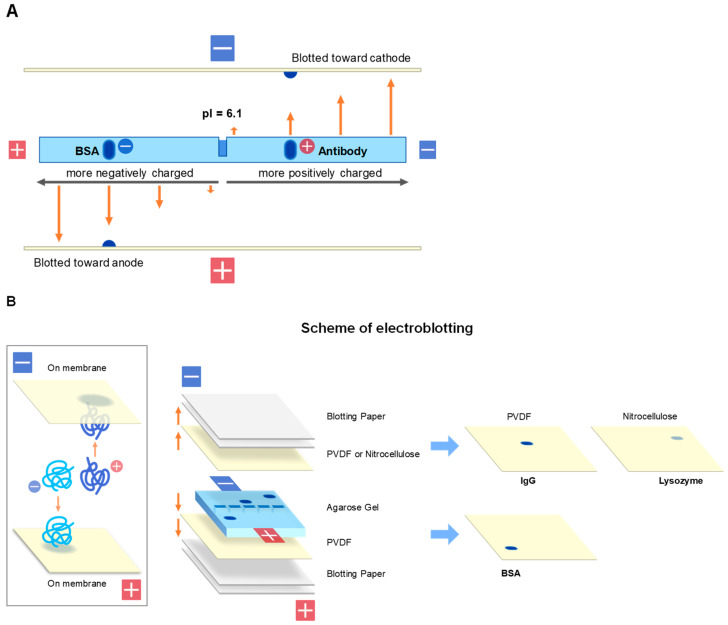
Schematic illustration of electroblotting from agarose gel. (**A**) Relationship between the net charges and direction and transfer efficiency in electroblotting. Indicated by arrows are hypothetical transfer efficiency due to the number of net charges. (**B**) Schematic illustration of native protein in blotting (inset) and actual setup of the blotting. Typically, electroblotting (semi-dry) was performed at a constant current of 1.5 mA/cm^2^ for 30 min.

Western blotting, shown in Figure 5 as one of the most important applications of gel electrophoresis, normally implies electroblotting from SDS-PAGE gels [43,44,45]. However, there are several blotting methods available to transfer proteins from the SDS-PAGE gels. Western blotting is most frequently used and is based on the negative charges of SDS-bound protein bands separated by SDS-PAGE. They are electrically transferred (i.e., electrophoresed) from the gel to the membrane placed on the anode side, as illustrated in Figure 7. In this blotting, proteins are fully denatured upon SDS-PAGE (see inset) by SDS micelle binding and blotted to PVDF or nitrocellulose membranes placed at the anode side of electrodes as depicted in Figure 7. Other transfer methods (e.g., diffusion blotting and vacuum blotting) are also used, although less frequently. The mechanism and efficiency would be significantly different for blotting of agarose native gels from the blotting of SDS-PAGE gels. On agarose native gels, protein bands are differently charged based on their amino acid sequences both in sign and magnitude. As schematically depicted in Figure 8, some proteins may be negatively charged and others may be positively charged on the gels. In addition, some proteins may be heavily charged, while other may be little charged (or uncharged). Depending on the charged states, some proteins may be transferred to the membrane on the anode side, as in Western blotting of SDS-PAGE, while others may be transferred to the membrane on the cathode. Efficiency of the transfer could depend on the degree of net charges at the pH of the transfer. Here, we describe three transfer methods we adopted and emphasize importance of maintaining the native structure during the blotting process.

### 2.1. Electroblotting

Electroblotting is the same as the Western blotting of SDS-PAGE. The net charge of the protein bands on the agarose gels is the driving force for transfer to the membranes. The major difference is the direction of the transfer, as the protein bands are charged based on their amino acid sequences and hence the isoelectric points. Positively charged proteins are theoretically transferred to the cathode side, while negatively charged proteins are transferred to the anode side, as depicted in Figure 8. In this electroblotting, a major difference occurs on transfer of basic proteins from blotting of negatively charged proteins from SDS-PAGE. As shown in Figure 8A,B, these basic proteins are positively charged and hence electrically transferred (electrophoresed) to the PVDF membrane on the cathode. We have developed the protocol to transfer proteins from agarose gels to membranes placed in both anode and cathode sides, as schematically depicted in Figure 8B where PVDF membranes are placed on both sides of the electrodes. However, we have noticed that lysozyme was not effectively transferred to the PVDF membrane at the cathode side. When nitrocellulose membrane was used at the cathode side, lysozyme transfer was effective, suggesting that highly basic proteins, such as lysozyme, may not be well bound to PVDF membrane and nitrocellulose membrane is more suitable.

### 2.2. SDS-Electroblotting

**Figure 9:**
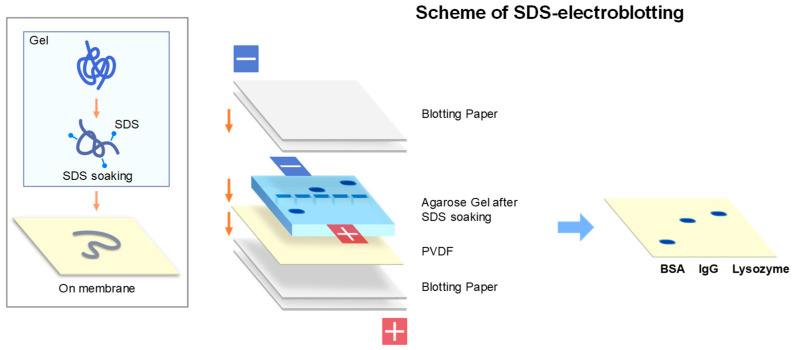
Schematic illustration of SDS-electroblotting. In inset is shown the structure of native protein that unfolds upon exposure to SDS below CMC. Right panel shows actual setup of SDS-soaked agarose gel in SDS-electroblotting. Blotting (Semi-dry) was typically performed at a constant current of 1.5 mA/cm^2^ for 30 min.

**Figure 10:**
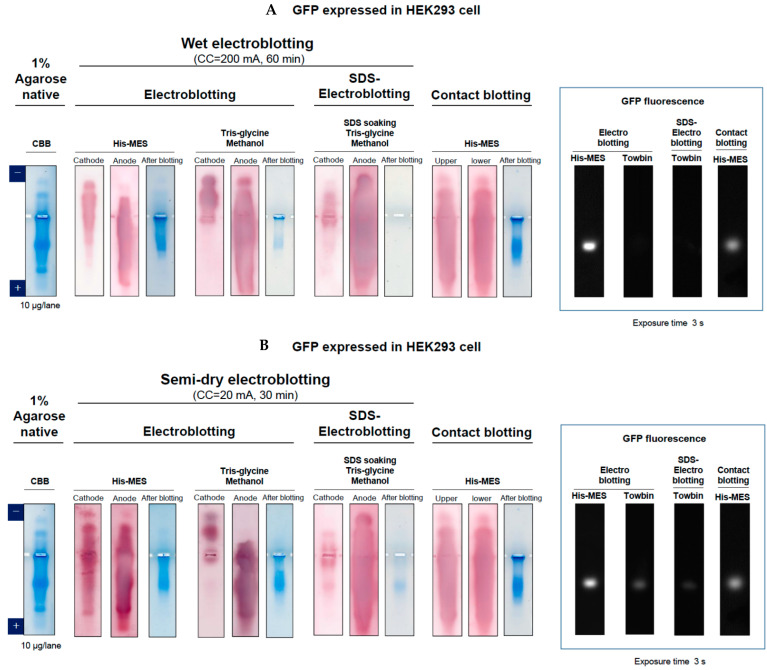
Comparison of electroblotting, SDS-electroblotting and contact blotting using HEK293 cell lysate expressing GFP and GFP fluorescence. (**A**) Wet blotting. (**B**) Semi-dry blotting. Transfer buffer: His/MES (pH 6.1), Tris-Glycine/Methanol (Towbin, pH 8.3). In SDS-electroblotting, agarose gels were soaked in 0.05% SDS prior to blotting in Towbin buffer. Blots were stained by Colloidal Gold Total Protein Stain. Agarose gels of pre-blot and post-blot were stained by CBB. These blots were compared for functional fluorescence state of GFP upon transfer (inset).

In this SDS-electroblotting, the standard protocol is to soak the agarose gel with 0.05–0.1% SDS. This does not fully mimic the event that happens on proteins during SDS-PAGE. On SDS-PAGE, protein samples are normally completely denatured by SDS concentration above 1%, namely above its CMC, as depicted in Figure 7. In this approach, we used 0.05–0.1% SDS to treat proteins on agarose gel to render them negatively charged, but not necessarily fully denatured, since such a low SDS concentration below CMC confers only molecular, not micellar, binding of SDS to the proteins, as depicted in Figure 9 (inset). Molecular SDS binding may be insufficient to cause complete protein denaturation. Then, just as in Western blotting, the agarose gel is subjected to electrotransfer, commonly referred to as “SDS-electroblotting”. The typical results are shown in Figure 10 with high efficiency. In Figure 10, PVDF membranes were placed on both sides of electrodes, while SDS-bound proteins are expected to be transferred only to the anode side, as depicted by membrane configuration in Figure 9. However, it turned out that the transferred proteins on the blot membrane are denatured, as demonstrated by non-fluorescence of GFP and no-detection by conformation-specific antibodies.

### 2.3. Contact Blotting

**Figure 11:**
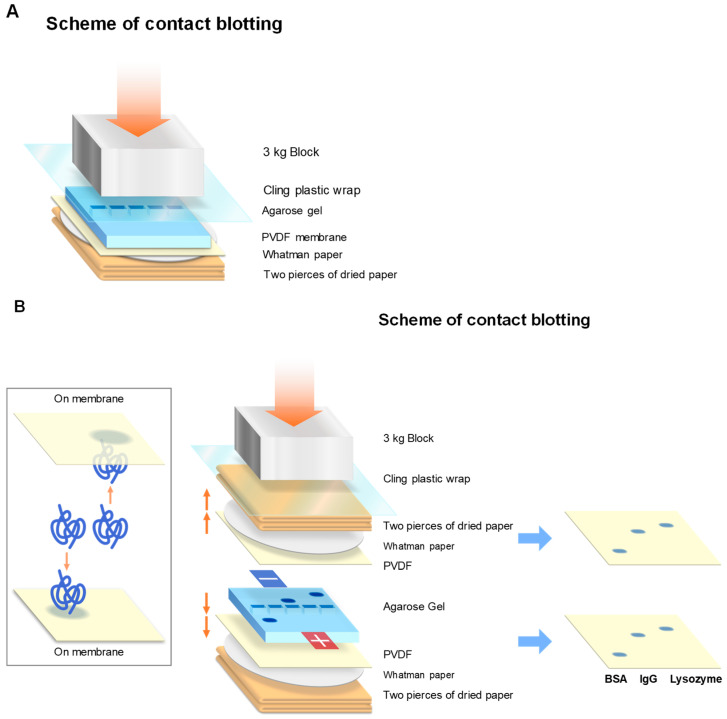
Scheme of contact blotting. (**A**) Contact blotting to PVDF membrane toward the bottom side. (**B**) Contact blotting to both upper and lower PVDF membranes. Typically, contact blotting was performed at room temperature for 5 min.

**Figure 12:**
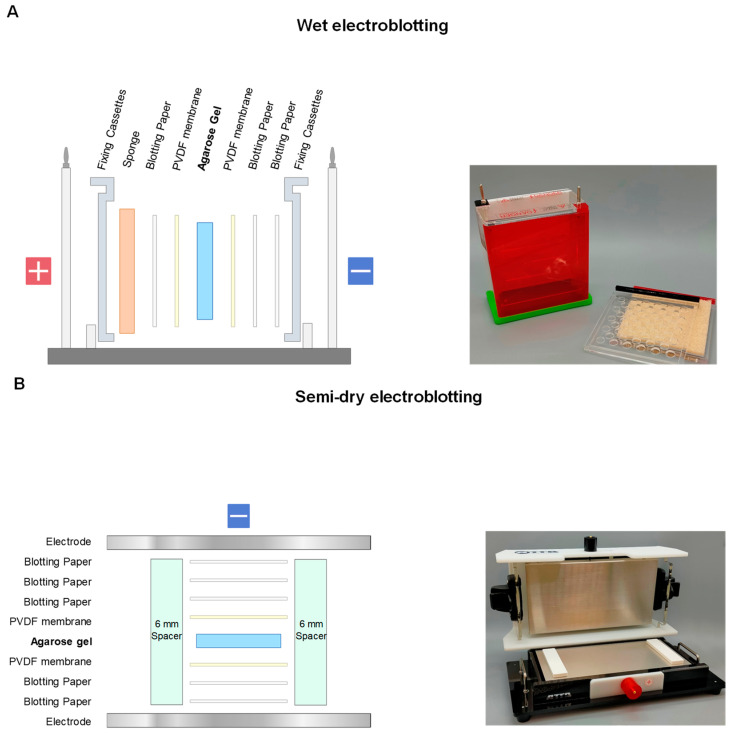
Comparison of blotting method. (**A**) Setup of wet blotting for electroblotting and SDS-electroblotting, where the entire setup is immersed in the transfer buffer. (**B**) Setup of semi-dry blotting for electroblotting and SDS-electroblotting, where PVDF membranes and blotting paper are soaked with the transfer buffer and spacers (6 mm) are placed on both sides to reduce the pressure due to the weight of the blotting device lid and thereby minimize protein diffusion before commencing electrotransfer process.

To avoid the above complication due to denaturation, we have developed contact blotting, which was adopted from Southern blotting [46] and modified for our purpose. The detail protocol has been described in [47]. The procedure is schematically depicted in Figure 11. In brief, agarose gel is sandwiched by two dry papers plus PVDF membrane and a plastic wrap to generate one blotted membrane, as depicted in Figure 10A (for one side blotting). Unless gravity is essential for contact blotting, it may be possible to generate two identical blots by placing the PVDF membranes on both sides of an agarose gel by symmetrically organizing dry papers and PVDF membranes, as depicted in Figure 11B (for two side blotting). It appears that gravity plays an insignificant role in transfer efficiency (Figure 12).

### 2.4. Comparison of Different Blotting

The above blotting was examined using cell lysates. The cell lysate was subjected to agarose native gel electrophoresis and stained by CBB or blotted using the above three blotting methods (i.e., electroblotting, SDS-electroblotting, and contact blotting). The electroblotting was performed under wet (Figure 12A, wet electroblotting) or semi-dry condition (Figure 12B, semi-dry electroblotting). Contact blotting was also carried out in both conditions with the setup shown in Figure 11B. Under wet condition of electroblotting, agarose gel was sandwiched by two PVDF membranes and filter papers in a horizontal mode, as depicted in Figure 12A. A sponge was inserted to reduce a sudden pressure to the gel. The entire set was then subjected to blotting in transfer buffer. Under semi-dry condition of electroblotting, a similar setup was constructed in a vertical mode, but without the sponge, as depicted in Figure 12B. Spacers (6 mm) were placed on both sides to minimize the effect of contact blotting due to the weight of the lid and to stabilize the layered structure of the gel and membranes. Contact blotting, which has no polarity, was also carried out under both wet and semi-dry conditions using a similar setup to Figure 12A,B.

Figure 10A shows the results of wet blotting and the CBB-stained agarose gel (far left) of the cell lysate prior to blotting. It is evident that a majority of lysate proteins are acidic, migrating toward the anode. Next panel shows the results of electroblotting carried out in an His-MES buffer, in which blots were stained by Colloidal Gold Total Protein Stain and post-blot gels were stained by CBB. A trend is clear that basic proteins were transferred to the cathode and acidic proteins were transferred to the anode, as expected from their own net charges. A small number of proteins remained in the post-blot gel, indicating incomplete transfer by this blotting method. The same electroblotting was carried out in the Towbin buffer (Tris-Glycine plus 20% methanol, pH 8.3). The results are similar in trend that basic proteins and acidic proteins were transferred to opposite electrodes. It should be noted that the pH of Towbin buffer of pH 8.3 is different from the pH of agarose gel and hence His/MES buffer. In this pH 8.3 transfer buffer, proteins at the basic side of the pH 6.1 agarose gel could become negatively charged and hence transferred to the anode side, which appeared to be the case. A major difference between electrotransfer in His/MES and Towbin buffers is transfer efficiency. It appears that the latter transfer is more efficient, as seen little CBB staining of post-blot gel. This may be due to different pH between His/MES buffer and Towbin buffer or the presence of 20% methanol in Towbin buffer, or both. On the contrary, SDS-electroblotting gave a different picture. Few proteins were transferred to the cathode and almost all proteins were transferred to the anode, as expected from the negatively charged state of proteins conferred by bound SDS. Namely, SDS converted basic proteins to negatively charged structures. No apparent protein staining was observed on post-blot gel after SDS-electroblotting, consistent with its transfer efficiency. Finally, the last panel shows the results of contact blotting, by which acidic and basic proteins were equally transferred to both upper and bottom sides of the PVDF membranes. This is expected due to its transfer mechanism, which is diffusion. Diffusion should occur at both sides of the agarose gel, unless gravity plays a role. The CBB staining of post-blot gel showed significant staining, indicating that the transfer is not compete by this contact blotting.

The above four blotting methods (i.e., electroblotting in His/MES or Towbin buffer, SDS-electroblotting and contact blotting) were compared for their effects on protein structure after blotting. As shown in Figure 10A (boxed far right), the electroblotting in His/MES gave a GFP fluorescent band (see bright band on dark background), consistent with no denaturing factors involved in this blotting method. However, electroblotting in Towbin buffer resulted in no fluorescence, perhaps due to denaturing effect of methanol. As expected from SDS binding, SDS-electroblotting caused no fluorescence. Contact blotting gave a fluorescent band, as expected from the transfer mechanism, which is simple diffusion. Thus, both non-denaturing transfers (i.e., electroblotting without methanol or SDS and contact blotting) resulted in fluorescently functional GFP.

A similar, but somewhat ambiguous, result was obtained under semi-dry condition. Figure 10B shows the results with cell lysates and GFP. With electroblotting in His/MES buffer, basic proteins were transferred to both cathode and anode sides, slightly more to the cathode. Acidic proteins were also transferred to both sides. These results imply that negatively charged proteins were transferred to both cathode and anode sides, regardless of the charged state and the same is true for the positively charged basic proteins. The most likely explanation is that with semi-dry condition, initial contact in His/MES buffer system between the gel and PVDF membranes resulted in immediate diffusion into both sides of membranes before even commencing electroblotting. Some proteins remained in the post-blot gel. The incomplete protein transfer was the case in electroblotting with Towbin buffer as well. However, this semi-dry blotting in Towbin buffer is identical to the wet blotting, as basic proteins were transferred to the cathode and acidic proteins were transferred to the anode. It thus appears that methanol in Towbin buffer prevents the above initial fast diffusion transfer by the contact between the gel and PVDF membrane (which has been soaked with Towbin buffer). SDS-electroblotting was the same as the wet condition. The same is true for contact blotting. Namely, both SDS-electroblotting and contact blotting are not affected by two different procedures, wet and semi-dry. GFP fluorescence was similar to the wet condition, as shown in Figure 10B (boxed). Namely, under semi-dry condition, GFP was fluorescent on electroblotting with His/MES and contact blotting. Weak fluorescence was observed, however, with electroblotting with Towbin buffer and SDS-electroblotting, perhaps suggesting refolding of GFP upon blotting under semi-dry condition.

## 3. Blotting Examples

### 3.1. GFP

**Figure 13:**
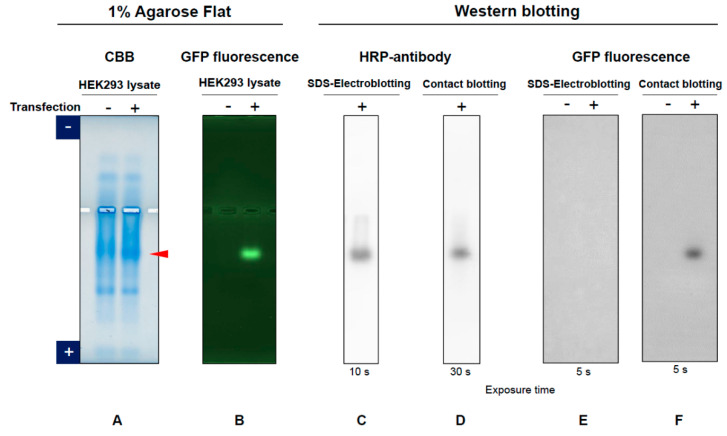
Comparison of SDS-electroblotting and contact blotting. In this blotting, PVDF membrane was placed only at the bottom side for contact blotting (pattern A in Figure 11) and at the anode side for SDS-electroblotting (semi-dry condition). (**A**) CBB staining of HEK293 cell lysates expressing GFP (+ lane). (**B**) GFP fluorescence on agarose gel. (**C**), GFP protein detected by HRP-conjugated antibody on SDS-electroblotting. (**D**) GFP protein detected by HRP-conjugated antibody on contact blotting. (**E**) GFP fluorescence on SDS-electroblotting. (**F**) GFP fluorescence on contact blotting.

Conformation of GFP after blotting was examined using contact blotting under semi-dry condition and SDS-electroblotting under wet condition. Figure 13 shows expression of GFP in the HEK lysate. The CBB staining showed an indication of GFP band with transfection (+), as shown by arrow. This band was fluorescent when illuminated by UV light (see green GFP fluorescence in + lane). The agarose gel was blotted by SDS-electroblotting or contact blotting and stained by HRP-antibody (horseradish-conjugated anti-GFP antibody). Both blotting methods gave a band, indicating successful transfer of GFP band from the agarose gel to the PVDF membrane. Namely, this antibody bound to both native (upon contact blotting) and denatured (SDS-electroblotting) GFP and hence is conformation-indifferential. However, only contact blotting gave a fluorescent band (see + lane of contact blotting), demonstrating that contact blotting resulted in transfer of native GFP on the blot and SDS destroyed the functional structure of GFP (see + lane of SDS-electroblotting). This also indicates that exposure of GFP to SDS even below CMC on agarose gel can denature the protein. Although not tested here, it is likely that electro-blotting would retain the functional GFP on the blot, as seen in Figure 10A,B (inset).

### 3.2. Anti-PLXDC2

**Figure 14:**
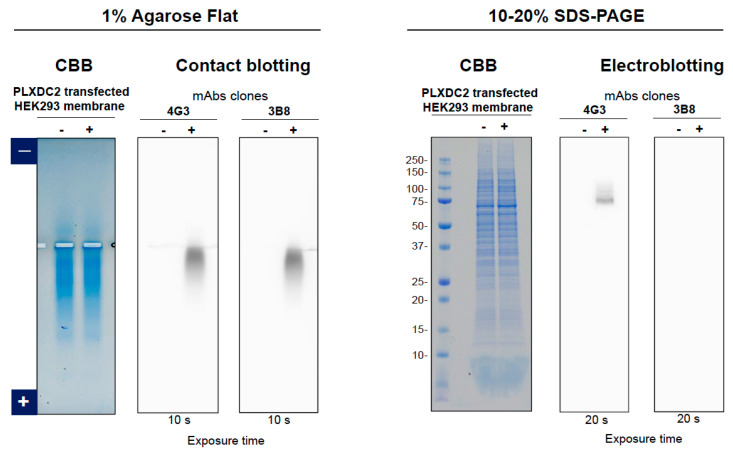
Comparison of contact blotting of agarose gel and electroblotting of SDS gel using PLXDC2 antigen. Left panel, 1% agarose gel and contact blotting. Right panel, SDS-polyacrylamide gel and electroblotting (semi-dry condition). For detection of human PLXDC2 protein, antibody 4G3 and 3B8 were used on both blots of agarose gel contact blotting and SDS polyacrylamide gel electroblotting.

Second example is the membrane fractions of HEK293 cells expressing a transmembrane human plexin domain containing protein 2 (PLXDC2). Figure 14 (left panel) shows the PLXDC2 antigen on PVDF membrane by contact blotting of agarose gel using two monoclonal antibodies, 4G3 and 3B8, which have been developed in-house to specifically bind to the antigen. CBB-stained agarose gel showed a similar pattern of HEK293 proteins. Figure 14 (right panel) shows the CBB-stained SDS-PAGE gel, indicating that the patterns are similar whether antigen was expressed or not. Figure 14 also shows binding of 4G3 and 3B8 to the denatured PLXDC2 antigen upon electroblotting of SDS-PAGE gel, where 4G3 bound to the denatured antigen. Thus, 4G3 is conformation-indifferential, namely binding to both native and denatured proteins. On the contrary, 3B8 showed no apparent binding to the SDS-denatured proteins, consistent with its conformation-specific properties.

### 3.3. Anti-SARS-CoV-2 Spike Protein

**Figure 15:**
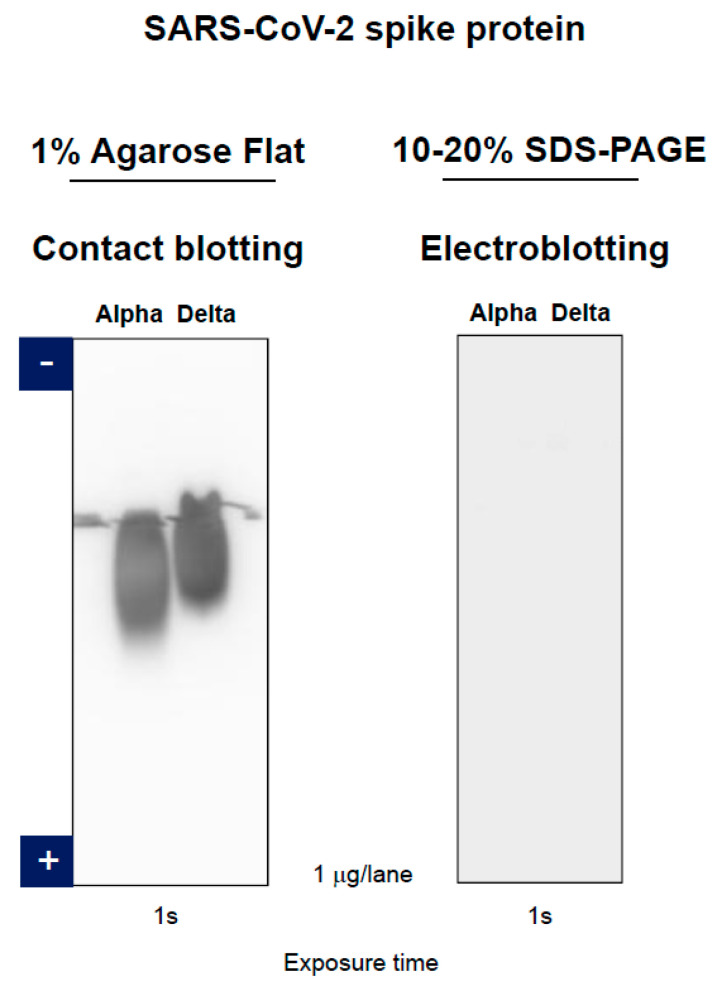
Comparison of contact blotting of agarose gel and electroblotting of SDS polyacrylamide gel using SARS-CoV-2 spike protein. Alpha and Delta variants of SARS-CoV-2 spike proteins were detected by specific monoclonal antibody. Left panel, contact blotting of agarose gel. Right panel, electroblotting of SDS polyacrylamide gel (semi-dry condition).

A similar observation was made with a humanized monoclonal antibody directed against SARS-CoV-2 spike protein. Figure 15 shows the contact blotting data for Alpha and Delta variants of SARS-CoV-2 spike protein detected by the monoclonal antibody. This antibody can bind to both variants when blotted from agarose gel by contact blotting, indicating that it does bind to the native antigen. On the contrary, it does not bind to both antigen variants transferred from SDS-PAGE gel by electroblotting, indicating that this monoclonal antibody is conformation-specific. Since it binds to native antigen of both variants, the results demonstrate that it should be able to detect both spike proteins on the virus surface.

## 4. Conclusions

Here, we reviewed three blotting methods (i.e., electroblotting, SDS-electroblotting, and contact blotting) and showed that agarose native gel elctrophoresis, when combined with electroblotting and contact blotting, leads to native, functional proteins on the blots. In addition, we showed that conformation-specific antibodies can be distinguished from conformation-indifferential antibodies by comparing the above native blotting with such denaturing blots as SDS-elecroblotting, electroblotting in methanol-containing transfer buffer and electroblotting of SDS-PAGE. We are now applying this technology for the analysis of protein complexes and complex mixtures of proteins and nucleic acids. When combined with native blotting, availability of conformation-specific and conformation-indifferential antibodies may shed light into the structural properties of component proteins derived from natural sources, including sera, tissues, cells, or plant products. In addition, these antibodies may be used as a ligand for orphan receptors, which may depend on their binding properties with the receptors. In general, conformation-specific antibodies may be expected to exert ligand-like function on the cell surface receptors.

## 5. Perspective

In this study, we reviewed potential applications of native proteins separated by agarose native gel electrophoresis. Such application potential may be furthered by increasing band sharpness, as a major drawback of this technology is broadness of the band shape due to porous nature of agarose gel with resultant diffusion of the proteins or their complexes. Among those applications, we consider 2D gel electrophoresis and blotting analyses of macromolecular complexes to be the most challenging and attractive. The highly porous nature of agarose gel may provide the advantage of separating macromolecular assemblies and transferring them to blot membranes or 2D gels.

## Data Availability

No new data were created or analyzed in this study. Data sharing is not applicable to this article.
